# Using Large Language Models to Enhance Exercise Recommendations and Physical Activity in Clinical and Healthy Populations: Scoping Review

**DOI:** 10.2196/59309

**Published:** 2025-05-27

**Authors:** Xiangxun Lai, Jiacheng Chen, Yue Lai, Shengqi Huang, Yongdong Cai, Zhifeng Sun, Xueding Wang, Kaijiang Pan, Qi Gao, Caihua Huang

**Affiliations:** 1 School of Sport Medicine and Rehabilitation, Beijing Sport University, No.48 Xinxi Road, Haidian District, Beijing, 100084, China; 2 Research and Communication Center for Exercise and Health, Xiamen University of Technology, 600 Ligong Road, Jimei District, Xiamen, 310204, China, 86 15606951380; 3 Department of Mathematics and Digital Science, Chengyi College, Jimei University, Xiamen, China; 4 School of Physical Education and Sport Science, Fujian Normal University, Fuzhou, China; 5 School of Marine Culture and Tourism, Xiamen Ocean Vocational College, Xiamen, China

**Keywords:** artificial intelligence, large language model, chatbots, exercise recommendations, physical activity, AI, LLM

## Abstract

**Background:**

Regular exercise recommendations (ERs) and physical activity (PA) are crucial for the prevention and management of chronic diseases. However, creating effective exercise programs demand substantial time and specialized expertise from both medical and sports professionals. Large language models (LLMs), such as ChatGPT, offer a promising solution by helping create personalized ERs. While LLMs show potential, their use in exercise planning remains in its early stages and requires further exploration.

**Objectives:**

This study aims to systematically review and classify the applications of LLMs in ERs and PA. It also seeks to identify existing gaps and provide insights into future research directions for optimizing LLM integration in personalized health interventions.

**Methods:**

A scoping review methodology was used to identify studies related to LLM applications in ERs and PA. Literature searches were conducted in Web of Science, PubMed, IEEE, and arXiv for English language papers published up to March 21, 2024. Keywords included LLMs, chatbots, ERs, PA, fitness plan, and related terms. Two independent reviewers (XL and CH) screened and selected studies based on predefined inclusion criteria. Thematic analysis was used to synthesize findings, which were presented narratively.

**Results:**

An initial search identified 598 papers, of which 1.8% (11/598) of studies were included after screening and applying selection criteria. Of these, ChatGPT-based models were used in 55% (6/11) of the studies. In addition, 73% (8/11) of the studies used expert evaluations and user feedback to assess model usability, and 45% (5/11) of the studies used experimental designs to evaluate LLM interventions in ERs and PA. Key findings indicated that LLMs can generate tailored ERs, save time in clinical practice, and enhance safety by incorporating patient-specific data. They also increased engagement and supported behavior change. This made PA guidance more accessible, especially in remote or underserved communities.

**Conclusions:**

This review highlights the promising applications of LLMs in ERs and PA but emphasizes that they remain a supplement to human expertise. Expert validation is essential to ensure safety and mitigate risks. Future research should prioritize pilot testing, clinician training programs, and large-scale clinical trials to enhance feasibility, transparency, and ethical integration.

## Introduction

### Background

Personalized, evidence-based exercise programs play a pivotal role in preventing and managing chronic conditions and reducing the risk of sports injuries. In contrast, poorly designed or inappropriate exercise practices can diminish effectiveness or even cause adverse effects  [1]. Therefore, formulating scientifically sound and effective exercise recommendations (ERs) is essential to optimize the health benefits of physical activity (PA) while mitigating potential risks. This approach is instrumental in improving chronic disease conditions and enhancing quality of life [2-4]. However, implementing these recommendations demands significant human resources and time.

Creating effective ERs requires nuanced, interdisciplinary collaboration between medical and sports professionals [5]. Medical experts, drawing on their knowledge of exercise physiology, medicine, and nutrition, consider individual health conditions, medical histories, and medication usage to ensure safety and efficacy. Simultaneously, sports professionals use their theoretical knowledge and practical experience to tailor individualized exercise plans based on skill levels and needs, working alongside medical counterparts to determine optimal recommendations.

Despite these collaborative efforts, the field of ERs has long faced a “black box” challenge, where the underlying mechanisms of exercise interventions are not fully understood, and outcomes are unpredictable [6-8]. Traditional approaches often rely on comprehensive strategies or personal intuition, introducing subjectivity and limitations. The advent of artificial intelligence (AI) in health care, however, offers promising insights into these complexities, enabling precise personalization.

AI’s robust data analysis, text generation, and creative capabilities are revolutionizing ERs and PA formulation, steering it toward precision and individualization. Notably, the linguistic understanding and generation abilities of large language models (LLMs), such as those based on transformer neural network architectures, have shown potential in personalized medicine and health management across various domains  [9-15].

Numerous studies have successfully tailored LLMs for specific domain tasks [16,17]. Research indicates that using self-supervision or continuing pretraining on domain-specific corpora enhances model performance in downstream tasks [18-20].

Given these developments, health and wellness are poised to be vital areas for AI’s future impact. By using advanced AI tools such as LLMs and chatbots, ERs and PA formulation is becoming increasingly scientific and personalized [21]. This not only has the potential to optimize exercise outcomes but may also enhance safety, ushering in a new era in chronic disease prevention and health management.

### Objectives

**Figure 1.**
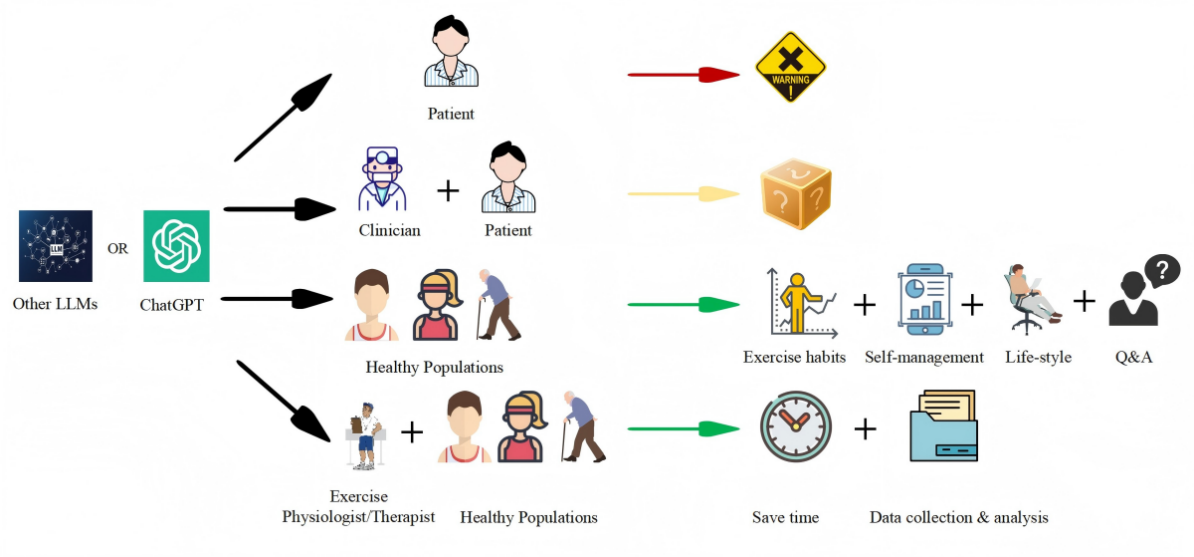
Potential roles and limitations of LLMs in exercise recommendations and physical activity. Direct use for patients is deemed inappropriate, while assisting clinicians requires further validation. LLMs have demonstrated benefits in promoting healthy habits and self-management among the general population. For exercise physiologists and therapists, LLMs enhance efficiency in client management, exercise suggestions, and data analysis. LLMs: large language models; Q&A: question and answer.

To our knowledge, this paper represents one of the first scoping reviews of the applications of LLMs in the fields of ERs and PA, with 2 primary objectives. First, we aim to summarize and categorize the applications of LLMs used in existing ERs and PA literature to identify trends, synergies, and patterns that could guide future investigations. Second, through our comprehensive review of the literature, we seek to provide valuable insights that can inform and shape the future development of AI technologies in the field of exercise health. By highlighting gaps and opportunities, we aim to assist in directing AI advancements to better meet the needs of personalized exercise and health management. This scoping review is unique not for its focus on health outcomes of interventions but for its emphasis on applying diverse LLM methodologies within the realms of ERs and PA and for providing guidance on future advancements in this interdisciplinary field (Figure 1).

The search strategy was designed to address several key research questions guiding this scoping review: (1) What is the current progress in applying LLMs for ERs in the health care field, and how effectively do LLMs support health care professionals in guiding patients toward personalized, evidence-based exercise program? (2) What is the progress of LLM applications in the PA domain for healthy populations, and can these models effectively assist in promoting personalized, evidence-based exercise program? (3) What methods have current studies used to validate the performance of LLMs, and do these models achieve the competency level of professional practitioners? (4) What challenges have been encountered in applying LLMs for ERs and PA, and what valuable insights do these challenges offer for future updates and iterations of LLM technology?

## Methods

### Study Design

We followed the scoping review methodology proposed by Arksey and O’Malley, which encompasses (1) identifying research questions, (2) relevant studies, (3) study selection, (4) data charting, and (5) collating, summarizing, and reporting the results. In addition, we adhered to the PRISMA-ScR (Preferred Reporting Items for Systematic Reviews and Meta-Analyses extension for Scoping Reviews) checklist to ensure comprehensive reporting.

### Search Strategy Development and Study Selection

The search strategy was developed through a collaborative process involving the research team members (XL and CH), who conducted the initial design and refinement of the search terms. The terms were informed by a thorough review of existing literature, discussions among the team members, and the key research questions identified at the outset of the study. These questions focused on the application and efficacy of LLMs in ERs and PA.

To ensure the robustness of the search strategy, we targeted 4 major databases: Web of Science, PubMed, IEEE, and arXiv, using comprehensive search strings that combined terms related to AI, LLMs, and exercise science. The search strategy included combinations of keywords such as “artificial intelligence,” “large language model,” “exercise recommendations,” and “physical activity” to capture a wide range of relevant studies.

While we did not formally consult a librarian or an information scientist, the team members leveraged their collective expertise in medical informatics and exercise science to craft a strategy that was both comprehensive and targeted. The detailed search process, including inclusion and exclusion criteria, is outlined in Table S1 in Multimedia Appendix 1.

### Eligibility Criteria

#### Inclusion Criteria

Non–peer-reviewed and non-English publications or resources were excluded. Studies were included in this review if they met the following criteria:

1. Language: Only those papers that were published in English were considered to ensure consistency and facilitate synthesis across studies.
2. Application of LLMs/Chatbot technologies: Studies that used LLMs or chatbot technologies in the context of ERs, PA guidelines, or fitness interventions were included.
3. Medical and public health context: Papers focusing on applications within medical practice or public health settings related to ERs, PA, or fitness were deemed eligible.
4. Diverse regions and health care contexts: Studies conducted across various geographic regions and health care settings to ensure global applicability and relevance of findings were included.

In addition, studies were considered if the terms “LLMs/Chatbots” and “ERs” or “PA” appeared simultaneously in the title, abstract, or keywords.

For classification purposes, (1) if the study population consisted of patients and the research focused on disease treatment, the study was categorized under ERs; (2) if the study population comprised healthy individuals and the research aimed at enhancing health levels, the study was categorized under PA, and (3) if these specific keywords did not appear but terms such as “fitness plan” or “exercise plan” were present, the study was included after discussion among all reviewers and then appropriately categorized.

#### Exclusion Criteria

Studies were excluded based on the following criteria:

1. Non–peer-reviewed and non-English publications: Publications that were not peer-reviewed or not published in English were excluded.
2. Nonoriginal research content: Reviews, abstracts, letters, viewpoints, editorials, dissertations, and tutorials were excluded unless they provided original research data.
3. Lack of relevant insights: Studies that did not offer substantial insights into the utilization of LLMs or chatbot technologies in ERs or related fields were excluded.

#### Data-Charting Process

A data-charting form was collaboratively developed by 2 reviewers (XL and CH) to determine which variables to extract. This form comprised six sections: (1) first author and publication date, (2) study design, (3) sample size or datasets, (4) related models, (5) usability testing, and (6) outcomes or contributions. The 2 reviewers (JC and SH) independently charted the data, discussed the results, and continuously updated the data-charting form in an iterative process.

To ensure the quality and relevance of the included papers, 2 reviewers (XL and CH) independently assessed the relevance of the papers to ensure a thorough and unbiased review process. In cases of disagreement, the remaining 4 authors (JC, ZS, YC, and SH) served as arbitrators, and a final decision was made only when consensus was reached among all 6 authors. To enhance consistency among reviewers, all reviewers initially screened the same publications, discussed the results, and amended the screening and data extraction manual before commencing formal screening. Working in pairs, the reviewers sequentially evaluated the titles, keywords, and abstracts of all publications identified in our searches for potentially relevant studies. Disagreements on study selection and data extraction were resolved through consensus and, if necessary, discussion with additional reviewers. This stringent selection process strengthens the validity and reliability of our findings by ensuring that our review is based on high-quality evidence.

#### Data Items

We abstracted data on paper characteristics (eg, first author, publication date, and study design), engagement characteristics and contextual factors (eg, sample size or datasets, and the specific LLMs or chatbot technologies used), barriers and facilitators to engagement (eg, results from usability testing and user interaction metrics), and results of any formal assessment of engagement (eg, key outcomes, contributions, and the impact of the models on ERs or PA).

## Results

### Identification of Studies

**Figure 2.**
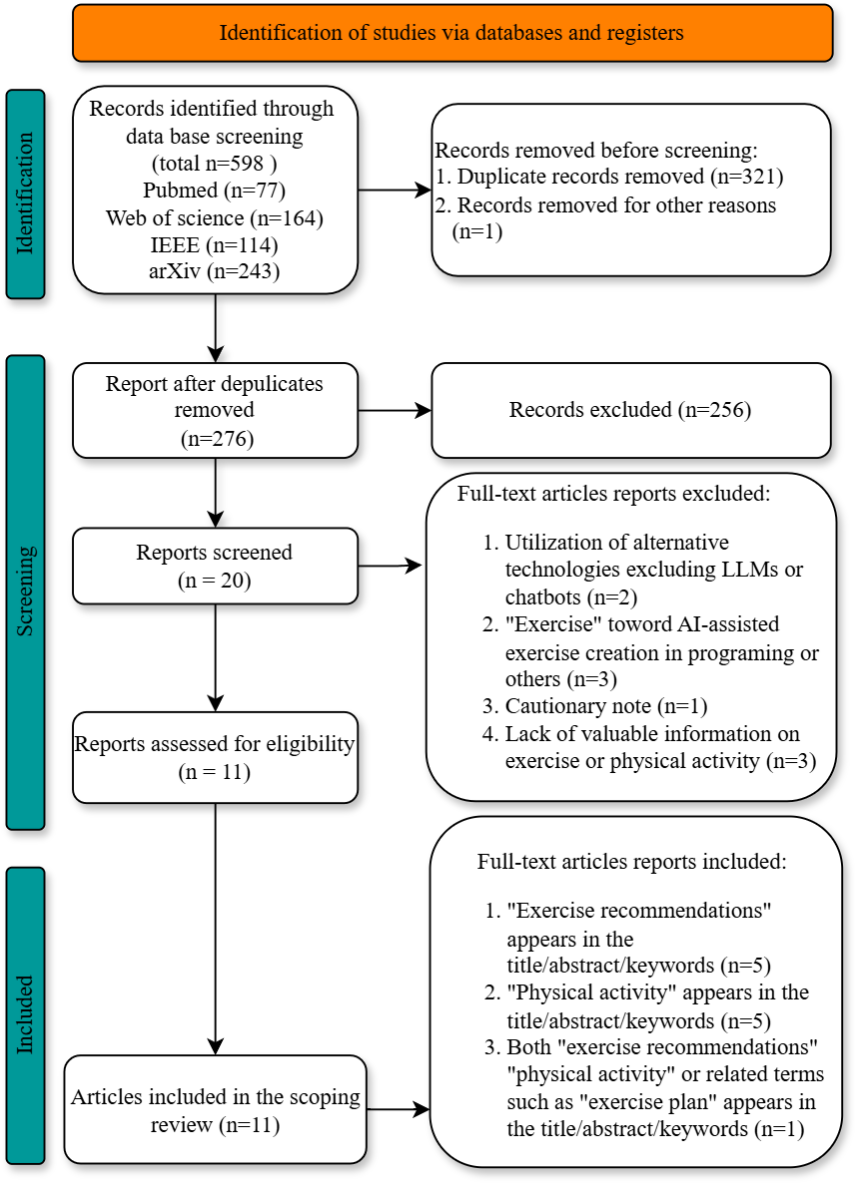
PRISMA-ScR (Preferred Reporting Items for Systematic Reviews and Meta-Analyses for Scoping Review) flow diagram. AI: artificial intelligence; LLMs: large language models.

An initial keyword search identified 598 papers, and after the removal of duplicates, 276 unique papers remained for screening based on their all fields. Of these, 256 papers were considered irrelevant and subsequently excluded from the review, one of the records cannot search full-text paper. After applying the study selection criteria to the remaining 20 papers, a total of 11 studies were included in the review (Figure 2).

### Study Characteristics

All the details regarding the study characteristics are comprehensively displayed in Tables1  2.

**Table 1.** Main characteristics of 5 reports focused on exercise recommendations in this review.

Author (year)	Study designs	Sample size or datasets	Related models	Usability testing	Outcomes or contribution
Zaleski et al (2024) [22]	Mixed methods study	N/A^a^	ChatGPT (February 2023 version)	Expert evaluations	Moderate comprehensiveness (41.2%) and high accuracy (90.7%).
Dergaa et al (2024) [23]	Simulated patient case study	5 profiles	GPT-4	Expert evaluation	Lack of precision for specific conditions; AI not yet a substitute for expert prescriptions.
Haag (2024) [24]	Experimental comparison	450 JITAIs^b^ decisions	GPT-4	User feedback	GPT-4-generated JITAIs rated highest in quality, effectiveness, and emotional impact, surpassing health care professionals and layperson-generated content.
Shin et al (2023) [25]	Exploratory study	18	LLM^c^-infused web interface	Expert evaluations.	AI^e^-generated exercise plans are personalized, actionable, and effective in supporting exercise routines.
Sivarajkumar et al (2023) [26]	Algorithm development and testing	13,605 patient records	Rule-based NLP^d^, ChatGPT	*F*_1_-score evaluation against gold standard dataset.	Rule-based NLP had best precision; ChatGPT had high recall and lower precision.

a N/A: not applicable.

b JITAIs: just-in-time adaptive interventions.

c LLM: large language model.

d NLP: natural language processing.

e AI: artificial intelligence.

**Table 2.** Main characteristics of 6 reports focused on physical activity in this review.

Author (year)	Study designs	Sample size or datasets	Related models	Usability testing	Outcomes or contribution
Willms and Liu (2024) [27]	Autoethnographic case study	N/A^a^	ChatGPT-3, Pathverse	Expert evaluations	Efficient content creation; recommended 6-step process for future mHealth^b^.
Chowdhury et al (2023) [28]	System development and evaluation	1M+ recipes	Neural network(Chatbot)	Empirical validation	Effective in recommending daily fitness and diet plans; future work aims to enhance personalization and usability.
Vandelanotte et al (2023) [29]	Conceptual framework	N/A	NLP^c^, ChatGPT^d^	User feedback	Developed a real-time framework for personalized interventions.
To et al (2021) [30]	Quasi-experimental study	116	Ida (Google Inc)	Self-reported	Significantly increased PA^e^ among participants.
Liang et al (2021) [31]	Randomized clinical trial	107 dialogues (7808 sentences)	BERT^f^-based classifier	Expert evaluations and user interaction analysis	Personalized dialogues reduced PA barriers, provided tailored support, and improved motivation for sustained PA behaviors.
Wiratunga et al (2020) [32]	Think-aloud methodology	7 participants	Voice-based chatbot (“FitChat”)	User feedback	Enhanced PA adherence and motivation through personalized, voice-based interactions.

a N/A: not applicable.

b mHealth: mobile health.

c NLP: natural language processing.

d GPT: Generative Pre-trained Transformer.

e PA: physical activity.

f BERT: Bidirectional Encoder Representations from Transformers.

### Main Findings

Five of the 11 studies reviewed used experimental designs, which constituted 45% [25,26,28,31,32]. These studies typically assessed the effectiveness of AI-driven interventions in ERs and PA, and ChatGPT-based models were used in 55% (6/11) of studies. In addition, 27% (3/11) of studies were case studies, focusing on real-world applications of chatbots and LLMs, while feasibility and development studies accounted for 27% (3/11) of studies, where prototypes and models were evaluated without full-scale trials [22,30,31].

### Sample Size or Datasets

Sample-based research: 5 studies (5/11, 45%) involved direct participant testing to assess the models’ effectiveness. These studies explored how LLMs could provide personalized recommendations for various populations [22-24].

Dataset-based research: 4 studies (4/11, 36%) used large datasets to train or fine-tune models, primarily focusing on improving the models’ functionality in specific use cases such as rehabilitation [26].

Other verification methods: 2 studies (2/11, 18%) used expert-driven or simulation-based evaluations rather than using participant data.

### Related Models

In the studies reviewed, ChatGPT-based models were used in 55% (6/11) of studies, with applications ranging from fitness recommendations to disease management. The remaining 45% (5/11) of studies used other LLMs or chatbot technologies, including Bidirectional Encoder Representations from Transformers–based models and specialized AI systems designed for specific interventions.

### Usability Testing

Expert evaluation: 6 studies (6/11, 55%) used expert evaluation as the primary method for usability testing. These evaluations typically involved health professionals or exercise specialists assessing the generated content or ERs for accuracy, relevance, and practicality [24,31].

Other methods: In 5 studies (5/11, 45%), alternative methods were used for usability testing, which included the following:

1. User interaction metrics: Studies monitored how participants interacted with AI-generated content, measuring factors such as engagement rates, adherence to ERs, and feedback from end users [22,23].
2. Real-time adaptive feedback: Some studies used just-in-time adaptive interventions, where the usability of the model was assessed based on how well it adapted to real-time changes in participants’ PA levels [24].

This mixed approach to usability testing highlights that while expert evaluations are crucial for initial validation, real-world interaction and feedback are also critical for assessing the practical application of the models in promoting ERs and PA.

### Outcomes or Contributions

Exercise recommendations: In total, 45% (5/11) of studies focused on generating ERs specifically within the medical domain. These studies revealed several key contributions:

1. Tailored interventions: The use of LLMs such as ChatGPT showed promise in generating personalized ERs based on patient health data and conditions [22-24,26,27]. Studies found improvements in accuracy when addressing clinical rehabilitation needs, particularly for cardiac and orthopedic rehabilitation [22,24].
2. Time saving in clinical practice: The integration of AI-driven ERs was shown to reduce the time needed for health care providers to develop exercise plans, improving efficiency in clinical settings [23].
3. Enhanced safety: By incorporating patient-specific health data, LLMs were able to create recommendations that accounted for safety concerns, especially for populations with chronic conditions [30,31].

Physical activity: In total, 55% (6/11) of studies focused on encouraging PA in healthy or general populations. Key contributions in this area include the following:

1. Increased engagement: LLMs and chatbots were effective at increasing PA levels by providing real-time feedback, adaptive exercise plans, and motivation through interactive features. This was particularly beneficial for populations such as older adults and sedentary individuals [23,30].
2. Behavior change support: AI-driven interventions helped users establish and maintain regular PA routines through personalized just-in-time adaptive interventions, demonstrating a positive impact on long-term behavior changes [24,25,28].
3. Accessibility improvements: Chatbots and AI systems provided low-cost, scalable solutions that increased accessibility to PA recommendations, particularly in remote or underserved communities [28].

## Discussion

### Principal Findings

The findings suggest that LLMs, such as ChatGPT, can effectively generate customized exercise plans, save health care professionals’ time, and enhance user engagement through adaptive, real-time feedback. However, it is crucial to emphasize that LLMs serve as a supplementary tool rather than a replacement for human expertise. Their outputs should always be reviewed and validated by qualified professionals, particularly in clinical settings where incorrect ERs could pose potential health risks [22-24,28]. These advantages are particularly beneficial in settings where access to personalized exercise guidance is limited. However, despite their potential, several key challenges and areas for future improvement have been identified.

### Comparison With Prior Work

The rapid advancement of LLMs has significantly impacted medical systems, offering enhanced adaptability and precision compared with traditional approaches. In specialized domains such as orthopedics, spinal disorders, and psychotherapy, LLMs demonstrate greater flexibility but still require refinement to meet professional health care standards [11-15].

Unlike previous static, rule-based systems, LLMs have extended their application to complex clinical scenarios such as cardiac rehabilitation and poststroke recovery [24-26]. Their ability to offer dynamic, real-time interactions marks a significant shift. However, they still fall short of professional-level expertise, particularly in aligning with the detailed exercise prescriptions outlined by American College of Sports Medicine guidelines [22,33].

A notable limitation in existing research is the variability in sample size and dataset quality. While some studies used extensive datasets to enhance model training and generalizability, others were constrained by limited sample sizes, which can hinder the robustness of the findings [24,26,34]. This inconsistency underscores the need for standardized, large-scale datasets to better assess and refine LLMs in ERs and PA contexts.

The current landscape of LLMs also features a variety of models, with many studies predominantly relying on ChatGPT [22-24,26,27]. This model shows strong capabilities in generating personalized ERs but remains supplementary to expert input. Comparatively, other models such as Bidirectional Encoder Representations from Transformers–based systems have been explored, offering unique strengths in specific applications [30]. Future research should explore and refine these models to optimize their use in exercise science, tailoring them to address the nuanced needs of this field [30,31].

Furthermore, usability testing remains a critical component in validating LLMs. While expert evaluations have been the primary method in many studies, incorporating real-world user feedback through interaction metrics and engagement analysis offers deeper insights into the practical use of these models. This dual approach—combining expert review with user-centric testing—ensures that LLMs not only meet theoretical standards but also deliver meaningful outcomes in everyday clinical practice [35].

Building on earlier AI applications, the integration of LLMs with wearable technology holds promise for transforming ERs and PA by delivering real-time, personalized insights. However, generative AI in exercise science still requires fine-tuning and specialized data to fully realize its potential. This underscores the ongoing need for high-quality, diverse data sources and iterative improvements to enhance the reliability and impact of LLM-driven health interventions [34].

LLMs significantly enhance patient engagement and satisfaction by delivering tailored, interactive, and context-aware health interventions. Through natural language understanding, LLMs such as ChatGPT facilitate real-time, adaptive interactions that make ERs and behavior change strategies more relatable and accessible, fostering deeper connections and prolonged participation in fitness routines [32]. Features such as just-in-time adaptive interventions ensure that guidance is provided at critical moments, reinforcing user commitment and fostering a sense of support while allowing users to dynamically adjust goals and promote sustained behavior changes [27]. Personalization is another key driver of satisfaction, as chatbots such as CHARLIE integrate fitness and diet suggestions into daily routines based on user-specific health data, schedules, and preferences, improving adherence and satisfaction [28]. However, sustaining engagement and satisfaction requires addressing challenges such as data accuracy, equitable access, and algorithmic biases. Transparent communication about data collection, robust validation mechanisms, and regulatory frameworks prioritizing inclusivity and fairness are crucial to maximize the potential of LLM-driven health interventions while ensuring scientifically grounded and emotionally supportive recommendations [29,31].

### Transparency of LLM Training Data and Fine-Tuning Approaches

One critical yet underexplored aspect of LLM development in ERs and PA is the lack of transparency regarding training data and fine-tuning methodologies. Most reviewed studies did not specify whether LLMs were trained or fine-tuned on exercise- or health-specific datasets, making it difficult to assess their domain relevance and reliability [22,24]. While some studies used general purpose LLMs, their applicability to exercise science remains uncertain due to potential biases and gaps in specialized knowledge [25,30,31].

To enhance technical rigor and reproducibility, future research should focus on documenting training data sources, improving dataset specificity, and incorporating domain-relevant corpora such as clinical guidelines, sports science literature, and patient-reported exercise data. In addition, developing benchmarking frameworks to systematically evaluate LLM-generated ERs will be essential for ensuring accuracy, safety, and adaptability in health care applications. Greater transparency in dataset composition and model fine-tuning will facilitate better comparisons across studies, enabling the optimization of LLMs for evidence-based exercise prescription.

### Limitations of the Study

Despite the valuable insights provided by this scoping review, several limitations should be acknowledged. First, the reviewed studies lacked transparency in data formats, training corpora, and methodologies, making it difficult to assess reproducibility and compare the effectiveness of different LLM implementations. In addition, none of the included studies evaluated the accuracy, safety, or long-term impact of LLMs on behavior change, limiting their clinical applicability. Second, small sample sizes and heterogeneous methodologies reduce the generalizability of findings [30]. Many studies focused on feasibility rather than efficacy, highlighting the need for large-scale, multicenter trials and long-term follow-up studies to validate AI-generated ERs [29]. Third, potential publication bias may have skewed the findings, as studies reporting positive outcomes are more likely to be published than those with neutral or negative results [22]. Future reviews should incorporate preprint archives and unpublished datasets for a more balanced assessment.

Finally, a lack of standardized datasets and evaluation criteria hinders direct comparisons between AI models [25]. Future research should focus on developing benchmark frameworks to ensure consistency and reliability in LLM-driven exercise prescription. Addressing these limitations will improve the transparency, safety, and clinical use of AI-assisted ERs .

### Future Directions

#### Real-World Clinical Trials

Future research should prioritize real-world clinical trials to address the current limitations of LLMs in health care, particularly in ERs and PA. Due to the reliance on synthetic data for training, many LLMs lack the real-world data necessary for optimal performance [9-15]. These trials are crucial for collecting high-quality data, enabling better fine-tuning of LLMs and ensuring that their recommendations are accurate and applicable across diverse populations. In ERs and PA, real-world trials can validate the practical effectiveness of LLMs, assess their generalizability, and ensure that their integration into clinical practice is both feasible and scalable, ultimately enhancing personalized health interventions [35] .

#### Fine-Tuning Existing Models

Currently, no model matches the expertise of exercise science professionals, largely due to inadequate corpus quality and computational limitations [24,32]. The field of exercise science particularly suffers from a lack of standardized data, with many practitioners relying on personal experience or generalized approaches to develop ERs [36]. While the notion that “one set fixed all” is prevalent, it falls short of delivering truly personalized and scientifically grounded exercise guidance. Therefore, the standardization of data formats and the accumulation of a vast array of high-quality data are essential steps for advancing generative AI’s capabilities in exercise science [37-39].

#### Potential of Retrieval-Augmented Generation

Retrieval-augmented generation (RAG) integrates the real-time retrieval of information with the generative capabilities of LLMs, offering a significant advantage in the domain of ERs and PA [12,28]. This approach ensures that any knowledge gaps in the language model are promptly addressed, reducing the risk of misinformation and enhancing the accuracy of exercise guidance [40]. The effectiveness of RAG is, however, contingent upon the underlying model’s strength, suggesting that fine-tuning a powerful language model in conjunction with RAG could optimize performance [41]. Looking forward, the application of RAG in ERs and PA could extend into broader health care sectors, potentially revolutionizing personalized medicine and proactive health management by providing data-driven, contextually rich, and adaptive health solutions.

#### Real-Time Data Collection Via Wearables and Smart Devices: Implementing a Dynamic Feedback Mechanism

The advent of wearable technology and smart devices has opened new horizons for monitoring health and PA in real time [21,22,34]. These devices, ranging from smartwatches and fitness bands to specialized sensors, continuously track physiological and PA data, including heart rate, steps, calories burned, sleep patterns, and advanced metrics such as heart rate variability and oxygen saturation. Their integration into health care enables real-world, individualized health monitoring, offering valuable insights for ERs interventions [22,25,42].

A key feature of this future direction is the establishment of a dynamic feedback loop between users and AI systems . As users engage in prescribed exercises, performance and physiological data are continuously fed into the AI model [43]. This real-time input allows LLMs to adjust recommendations dynamically, enhancing adherence and optimizing exercise effectiveness [24,30].

However, several technical challenges remain. Data accuracy and reliability vary due to sensor inconsistencies, calibration differences, and environmental influences [28]. Standardized validation protocols and AI-driven error correction models are needed to enhance measurement consistency. In addition, device compatibility and interoperability pose integration hurdles, as wearables operate with different data formats and application programing interfaces [32]. Adopting universal data standards, such as Fast Healthcare Interoperability Resources and Open mHealth, could streamline integration across platforms.

**Figure 3.**
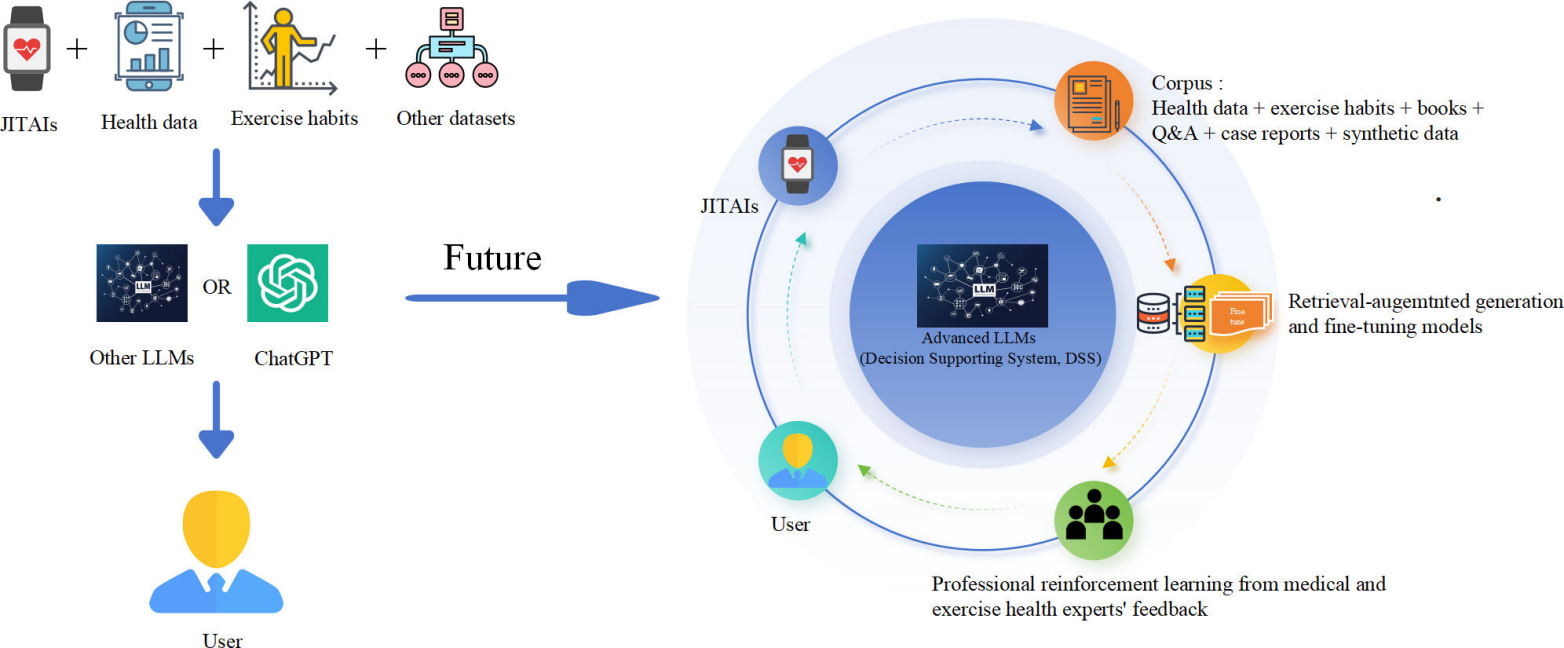
The current and future applications of LLMs in exercise recommendations. Currently, health data from JITAIs, wearable devices, exercise habits, and various datasets are processed through LLMs such as ChatGPT to provide recommendations to users. Looking forward, advanced LLMs integrated within a Decision Support System (DSS) will use retrieval-augmented generation and fine-tuning techniques based on extensive corpora including health data, exercise habits, literature, Q&A content, case reports, and synthetic data. Enhanced by real-time data collection via wearable devices and continuous professional reinforcement learning from medical and exercise health experts' feedback, this system aims to support personalized recommendations while addressing critical issues such as clinical validation, integration into health care workflows, personal privacy, artificial intelligence bias, and ethical and regulatory considerations. JITAIs: Just-in-Time Adaptive Interventions; LLMs: large language models; Q&A: question and answer.

Data privacy and security are also critical concerns, given the continuous transmission of personal health information. Compliance with health insurance portability and accountability act, general data protection regulation, and other regulatory frameworks is essential to prevent data breaches and unauthorized access [26]. End-to-end encryption, federated learning, and decentralized storage solutions can enhance security while maintaining user privacy. Addressing these challenges will be key to ensuring the safe and effective deployment of LLMs in personalized exercise interventions (Figure 3).

#### Expanding Clinical Validation of LLMs

Despite the growing interest in LLM-driven ERs, real-world clinical validation remains limited, with most studies focusing on feasibility rather than long-term efficacy and safety [22]. To bridge this gap, emerging trials are exploring LLM-assisted exercise coaching in rehabilitation [25] and AI-driven personalized exercise plans for metabolic disorders [28].

However, large-scale, multicenter randomized controlled trials are needed to assess their impact on exercise adherence, rehabilitation outcomes, and chronic disease management across diverse populations. Future research should prioritize standardized evaluation metrics, regulatory oversight, and expert-in-the-loop models to ensure safe, effective, and evidence-based integration of LLMs into health care practice.

#### Integrating LLMs Into Health Care Workflows

Effective integration of LLMs into clinical practice requires interoperability with electronic health records (EHRs) and clinician training [35]. Standardizing LLM-EHR interactions through protocols such as Fast Healthcare Interoperability Resources and HL7 can enable real-time data exchange and personalized exercise prescription delivery [23]. In addition, structured clinician training programs are essential to enhance AI literacy, ensuring that health care providers can interpret, validate, and oversee LLM-generated recommendations before clinical application [43]. Implementing expert-in-the-loop frameworks, where clinicians supervise AI outputs, will enhance trust, safety, and adoption in real-world health care settings.

#### Personal Privacy, AI Bias, and Ethical and Regulatory Considerations

Ensuring robust data privacy and security measures is critical in the deployment of LLMs in health care, as secure data management is essential to protect sensitive health information and safeguard users’ personal privacy [28,32]. Maintaining patient confidentiality, particularly when using EHRs in precision medicine, remains a persistent challenge that requires systematic attention [25,26]. Addressing AI bias is equally crucial to prevent inequities in rehabilitation plans and ensure fair and inclusive recommendations across diverse demographic groups, especially in the context of ERs and PA. For example, general purpose LLMs may produce exercise suggestions that are overly strenuous for older adults or unsuitable for individuals with chronic conditions, thereby increasing the risk of injury or adverse health outcomes [25,28,30]. Strategies to mitigate bias include implementing advanced detection algorithms, establishing fairness metrics tailored to the exercise and health domains, and using diverse and representative datasets that encompass a wide range of ages, genders, races, and socioeconomic backgrounds. Moreover, fine-tuning LLMs with domain-specific data and incorporating expert insights from exercise physiologists can further tailor recommendations to the unique needs of these populations [22]. Incorporating feedback loops from underrepresented populations further refines model outputs, enhancing the inclusivity and effectiveness of LLM-driven interventions [16,17].

Transparency and ethical accountability play a foundational role in building user trust and fostering the successful adoption of LLMs in health care. Clear communication about data usage practices, system limitations, and decision-making processes is imperative to enhance user confidence, as a lack of transparency may discourage full engagement with AI-driven tools, ultimately reducing their effectiveness [28]. Furthermore, LLMs face significant ethical challenges, including the potential to provide inaccurate or unsafe recommendations, which could result in adverse health outcomes. Integrating expert oversight and providing explicit disclaimers about the limitations of LLM-generated advice are essential safeguards to mitigate such risks [27]. Finally, compliance with evolving regulatory standards is imperative. LLM-based systems must adhere to rigorous requirements for accuracy, reliability, and accountability to align with medical device regulations and ethical norms, ensuring their safe and effective integration into health care practices [29]. This holistic approach to privacy, bias mitigation, transparency, and regulatory compliance will not only enhance the fairness and accuracy of LLM-driven health interventions but also establish a foundation of trust necessary for their widespread acceptance and long-term success.

### Conclusions

While LLMs offer promising support in creating exercise plans, they are not yet on par with expert professionals. Their role should be viewed as an adjunct to human expertise, assisting but not replacing health care and fitness professionals. Ensuring expert validation is essential to mitigate risks and optimize patient safety. Future research should prioritize pilot studies to assess the feasibility of LLM-generated ERs and clinician training programs to enhance AI literacy and integration.

Advancing LLM adoption requires large-scale trials, standardized evaluation frameworks, and regulatory oversight to improve transparency and mitigate bias. Leveraging real-time data from wearables can further refine recommendations. With these advancements, LLMs can evolve into a valuable decision support tool, enhancing accessibility and personalization in exercise science while maintaining expert oversight.

## Supplementary material

10.2196/59309 Multimedia Appendix 1 The search strategy and detailed summary.

10.2196/59309 Checklist 1 PRISMA-ScR (Preferred Reporting Items for Systematic Reviews and Meta-Analyses extension for Scoping Reviews) checklist.

## Abbreviations

AI: artificial intelligence
EHR: electronic health record
ER: exercise recommendation
LLM: large language model
PA: physical activity
PRISMA-ScR: Preferred Reporting Items for Systematic Reviews and Meta-Analyses extension for Scoping Reviews
RAG: retrieval-augmented generation
