# Supplementary material for: Using Large Language Models to Enhance Exercise Recommendations and Physical Activity in Clinical and Healthy Populations: Scoping Review
Source: JMIR Med Inform. 2025 May 27;13:e59309. doi: 10.2196/59309 (PMC12133071; doi:10.2196/59309)
Supplement: Multimedia Appendix 1 [file medinform-v13-e59309-s001.docx]

**Table S1.** The search strategy and detailed summary.

| Items | Specification |
| --- | --- |
| Date of search | Mar. 21 st, 2024 |
| Databases searched | Web of Science, PubMed, IEEE, and arXiv |
| Search strategy | 1. TS= (“artificial intelligence” OR “AI” OR “neural network*” OR “machine learning” OR “multimodal” OR “transfer learning” OR “interpretability” OR “Transformer”) 2. TS= (“Large language model*” OR “Natural Language Processing” OR “NLP” OR “GPT” OR “LLMs” OR “chatbot*”) 3. TS= (“exercise” OR “exercise recommendations” OR “exercise recommendations” OR “physical recommendations” OR “physical activity” OR “physical exercise” OR “fitness”) 4. #1 AND #2 AND #3 |
| Inclusion criteria | 1. Problems: Focus on the application of exercise recommendations, physical activity, or fitness in the context of medicine or public health 2. Intervention: interventions via LLMs/Chatbots 3. Comparison: LLMs or Chatbots in practical exercise recommendations, physical activities settings or test their capability in this domain 4. Outcomes: reporting of intervention impact on participants or participants’ experiences with the conversational agent; some description of theoretical basis, dialog flow development, or intervention components of the program 5. Study type: Restricted to English articles |
| exclusion criteria | 1. Problem: studies that did not target LLMs/Chatbots for exercise recommendations, physical activity in users 2. Intervention: interventions that did not involve LLMs/Chatbots 3. Comparison: without empirical application or testing related to exercise recommendations and physical activities 4. Outcomes: no mention of intervention impact or participant experiences; no description of the applied intervention 5. Study type: reviews, encompassing abstracts, letter, viewpoint, Editorials, dissertations, tutorials and restricted to English articles |
| Selection process | Two authors, Xiangxun Lai and Caihua Huang, independently review for thematic relevance. In case of disagreement, four more authors Chen, Sun, Cai and Huang, serves as arbitrators, and the decision for inclusion is made only when all of the authors are in an agreement. |
